# Tissue oxygenation as a target for goal-directed therapy in high-risk surgery: a pilot study

**DOI:** 10.1186/1471-2253-14-122

**Published:** 2014-12-16

**Authors:** Paul A van Beest, Jaap Jan Vos, Marieke Poterman, Alain F Kalmar, Thomas WL Scheeren

**Affiliations:** Department of Anesthesiology, University Medical Center Groningen, University of Groningen, PO Box 30001, 9700 RB Groningen, the Netherlands

**Keywords:** Tissue oxygenation, Near-infrared spectroscopy, Postoperative complications, High-risk surgery, Goal-directed therapy

## Abstract

**Background:**

Tissue hypoperfusion occurs frequently during surgery and may contribute to postoperative organ dysfunction. There is a need for perioperative treatment protocols aiming at improving tissue oxygenation (StO_2_). We hypothesised that intra-operative optimisation of StO_2_ improves tissue perfusion and thus reduces postoperative complications. Furthermore, we evaluated the feasibility of the optimisation algorithm used.

**Methods:**

We randomized 50 high-risk patients, all >65 years with ASA physical status III, who underwent major abdominal surgery under standardized balanced general anesthesia combined with epidural analgesia. Throughout surgery StO_2_ was monitored at the thenar eminence using near-infrared spectroscopy. All patients were treated according to a standard care algorithm. In addition, patients in the intervention group were treated with dobutamine if necessary to keep or raise StO_2_ ≥ 80%. Data were recorded continuously and complications were recorded during hospital stay with a maximum of 28 days.

**Results:**

The number of complications was not significantly different between groups (11 vs 20; p = 0.23). Eleven patients in the intervention group had no complication, versus 7 in the control group. There was no significant difference between groups in length of stay in ICU or in hospital. Only ten patients in the intervention group received dobutamine. Administration of dobutamine resulted in a moderate 6 [-3 to 10] % increase of StO_2_. The overall protocol adherence was 94%.

**Conclusions:**

No statistically significant difference in outcome was realized through intraoperative optimization of StO_2_ values in this pilot study. The protocol used may be considered feasible for clinical practice. Further research is obligatory to define both the optimal StO_2_ threshold and intervention to treat tissue hypoperfusion.

**Trial registration:**

ClinicalTrials.gov identifier: NCT01342900. Registered 21 April 2011.

## Background

Postoperative complications determine long-term survival after high-risk surgery. Although high-risk surgical patients account for a minority of surgical procedures they represent a vast majority of post-operative mortality rates [1]. Therefore, a decrease in post-operative complications is important and can be achieved by using goal-directed fluid optimization [2]. In order to limit post-operative complications, adequate monitoring and optimisation of physiological variables during surgery are necessary.

Hemodynamic variables such as heart rate, arterial pressure and central venous pressure are commonly used but have been proven to be poor predictors of outcome. In contrast, the use of flow-related variables such as cardiac index (CI), oxygen delivery (DO_2_) and central venous oxygen saturation (ScvO_2_) have been associated with improved outcome after high-risk surgery [3]. Improvement of these hemodynamic and oxygen derived variables may affect tissue oxygenation and as a result preserve organ function. While these variables reflect global changes in either the upstream or the downstream part of the circulation and not regional changes as tissue oxygenation, they may be of vital importance since undetected tissue hypoxia may contribute to postoperative organ dysfunction. Remarkably, even though tissue hypoperfusion occurs frequently during high-risk surgery or in high-risk patients undergoing surgery [4, 5] there is a lack of protocols for the perioperative management of high-risk surgical patients [6].

The purpose of the present interventional pilot study is primarily to evaluate the effect of the use of a protocol based on optimisation of tissue oxygenation (StO_2_) on the incidence of perioperative complications. As a secondary objective, we assessed the feasibility of such optimisation protocol in clinical practice.

## Methods

The study complied with the Declaration of Helsinki. The protocol was approved by the Local Ethics Committee (Medisch Ethische Toetsingscommissie UMC Groningen; METc 2010/209) and the study was registered on ClinicalTrials.gov (NCT01342900). Written informed consent was obtained from all participants during the pre-anesthetic consultation.

### Patients

Eligible patients were ASA III and IV patients of 65 years and older who were scheduled to undergo elective major surgery under general anesthesia combined with epidural analgesia.

Exclusion criteria were patients undergoing emergency surgery, refusal, neurosurgical patients, and patients with disseminated malignancy or receiving palliative treatment only. Patients undergoing extensive liver surgery requiring low central venous pressure (CVP) management and patients scheduled for a fast track protocol were excluded as well.

### Anesthetic technique

The patients received a standardized balanced anesthesia. Each patient received acetaminophen 1000 mg orally as premedication. Standard vital signs monitors were placed, including Bispectral monitoring [BIS, Aspect Medical Systems, Norwood, USA]. Thoracic epidural catheter was placed by standard procedure. Anesthesia was induced using target-controlled infusion (TCI) of sufentanil with an effect site concentration (C_e_t) of 15 ng ml^-1^ and TCI of propofol (2–4 μg ml^-1^ C_e_t) according to institutional practice, adjusted if necessary to keep BIS in the range of 40–60. After loss of verbal contact and confirmation of adequate mask ventilation, rocuronium (0.6 mg kg^-1^) was administered and the patient’s trachea was intubated. Patients were mechanically ventilated (volume controlled mode) with a tidal volume of 7 ml kg^-1^ body weight, unless required otherwise, i.e. one lung ventilation during oesophagotectomy. Subsequently, TCI sufentanil was reduced to 0.10 ng ml^-1^ C_e_t. A bolus of 6–8 ml 0.25% levobupivacaine and 20–30 μg sufentanil was given epidurally before start of surgery, and at skin incision sufentanil was increased to 0.20 ng ml^-1^ C_e_t. Epidural analgesia with 0.125% levobupivacaine + 1 μg ml^-1^ sufentanil was maintained at a rate of 6–8 ml h^-1^. A radial or brachial arteria was cannulated for continuous measurement of the arterial pressure and was connected to the FloTrac Vigileo system (Version 03.02; Edwards Lifesciences LLC, Irvine, CA, USA) for measuring cardiac output (CO). All patients received a central venous line (jugular vein) with continuous oximetry (PreSep catheter; Edwards Lifesciences LLC, Irvine, CA, USA) for measuring ScvO_2_.

### Study protocol

**Figure 1:**
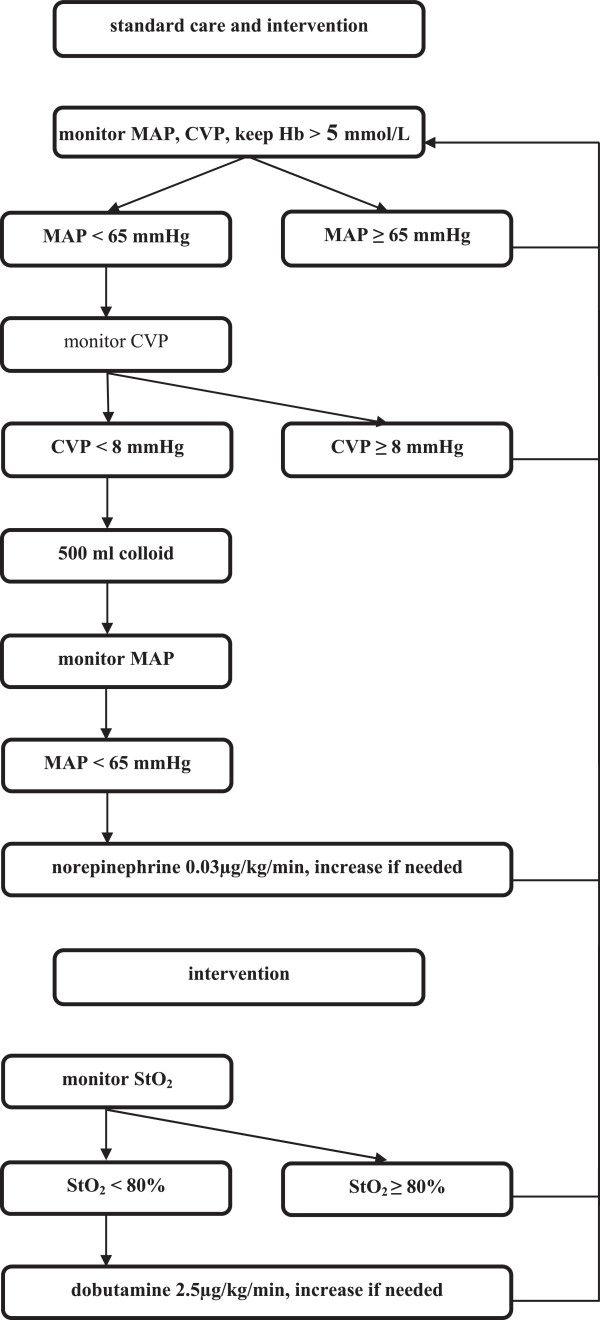
**Standard care and intervention algorithm.** MAP, mean arterial pressure; CVP, central venous pressure; Hb, hemoglobin; StO_2_, tissue oxygenation.

StO_2_ was monitored non-invasively in all patients by near infrared spectroscopy using the InSpectra System [Model 650; Hutchinson Technology, Hutchinson, MN, USA]. The device consists of a monitor and a sensor, which is placed on the thenar eminescence of the patient. In the control group the anesthetist/investigator was blinded for data from the InSpectra monitor. All patients were treated according to the standard care algorithm (Figure 1). No dobutamine was to be administered in the control group. In addition, patients in the intervention group were treated according to the intervention algorithm aiming to keep or raise the StO_2_ ≥ 80% (Figure 1). If the patient’s heart rate exceeded 110 beats per minute or if new rhythm disturbances occurred during dobutamine infusion it was left to the anesthesist’s discretion not to give or not to increase dobutamine.

#### Randomisation

After induction of anesthesia, but prior to the start of the surgery, patients were randomly assigned to the control group or the intervention group using sealed envelopes.

#### Follow-up

Upfront, all patients were scheduled to be postoperatively monitored either at an intensive care unit (ICU) or at a prolonged stay at the post-anesthesia care unit (PACU). All PACU and ICU teams were blinded for patients’ allocation. Follow-up for postoperative complications continued during the total hospital stay with a maximum of 28 days.

### Data collection

All standard data from the anesthesia monitor (MP70, Philips, Eindhoven, NL) was recorded continuously (sampling rate of 1 Hz) utilizing data-logging software (RugLoop II, Demed Engineering, Temse, Belgium) on a medical grade Windows XP based personal computer. The StO_2_ data of the InSpectra™ device and the hemodynamic variables were imported into Microsoft Excel 2010® (Microsoft, Redmond, WA, USA) for synchronisation and analysis. After graphical representation, a visual inspection of the data plots was performed to delete obvious atypical values caused by artifacts. Next, a 30-second moving median with 15-second steps was calculated for all studied variables. The evolution of the absolute values was plotted from 10 minutes before the start of dobutamine administration until 45 minutes thereafter.

All commonly used variables were recorded, including ventilator settings. In addition, patient characteristics, surgical information, fluid administration, and use of inotropes were collected. To prevent a ruffled picture on length of stay numbers due to the logistic reality of the PACU or the ICU, ‘fit for discharge’ criteria were noted every 6 hours during PACU/ICU stay. These criteria were: spontaneous breathing, oxygen saturation (SpO_2_) > 92% with < 3 litre O_2_/minute, systolic arterial pressure > 100 mm Hg without vasoactive or inotropic support, and temperature in range of 36–38.5°C. The patients were considered fit for discharge when all criteria were fulfilled. Both the therapeutic intervention scoring system (TISS)-score and sequential organ failure assessment (SOFA)-score were noted for insight in resource utilization and the degree of organ failure.

### Statistical analysis

A priori, the population was divided into two groups: control group vs. intervention group. In addition, a subgroup from the intervention group was analyzed, i.e. patients in whom dobutamine was actually used (Dobu^+^ subgroup). Two-tailed statistical tests were performed by the statistical package for the social sciences (IBM SPSS 20 for Windows, Chicago, IL, USA). GraphPad software (Prism 5.0, La Jolla, CA, USA) was used for graphics. All continuous data were tested for normal distribution with the D’Agostino-Pearson omnibus normality test before further statistical analysis. Differences between groups were assessed using Student’s *t*-test or Wilcoxon-Mann–Whitney test where appropriate. For categorical data Chi-Square test or Fisher’s exact test was applied. Statistical significance was assumed at p < 0.05. Non-parametric data are presented as median [quartiles]. G Power was used to calculate a sample size from differences in the number of post-operative complications between the two study groups [7].

## Results

### Patient characteristics

**Figure 2:**
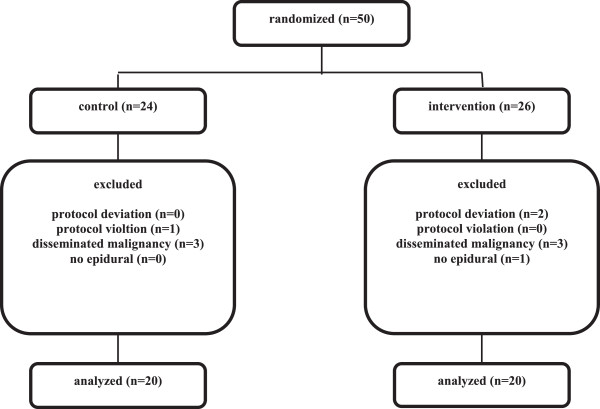
**Flowdiagram of the study.**

A total of fifty patients were included and randomized of whom ten patients had to be excluded from the study. The reasons for exclusion were protocol deviation (n = 2; poor use of equipment), protocol violation (n = 1; use of dobutamine in control group), meeting exclusion criteria peri-operatively (n = 7; disseminated malignancy or unable to place epidural catheter). As a result, forty patients were included for analysis (Figure 2).

Table 1 displays patient characteristics and intraoperative characteristics. Comorbidities were similar in both groups. All patients were scored grade III ASA physical status classification. No significant differences between the two groups with respect to type of surgery and administered fluids during surgery were noticed.

**Table 1:** **Patient baseline and intraoperative characteristics (n = 40)**

Characteristics	Control (n = 20)	Intervention (n = 20)	P-value*	Dobu ^+^ (n = 10)	P-value ^§^
Age (yr)	75 [69–78]	71 [69–78]		71 [69–81]	
Gender (M/F)	16/4	16/4		9/1	
Body Mass Index (m/kg^2^)	27 [22–31]	25 [23-27]		25 [23–28]	
Smoking status (%)	60	35		40	
Medical history (n)					
COPD	4	4		1	
CAD	5	9		5	
CHF	5	3		2	
IDDM	3	6		3	
TIA	2	2		1	
Hemoglobine (g/dl)	12.2 [10.6-14.5]	13.7 [12.6-15.3]	0.34	13.4 [10.6-15.6]	0.25
Type of surgery (n)			0.18		0.13
AAA	9	5		2	
Oesophagus	1	2		2	
Stomach	1	3		1	
Pancreas	1	5		4	
Colon	3	2		0	
Other	5	3		1	
Duration surgery (min)	277 [221–334]	262 [145–387]	0.63	349 [246–500]	0.35
Blood loss (ml)	800 [350–1575]	375 [200–950]	0.07	550 [288–1050]	0.22
RBC transfusion (ml)	0 [0–300]	0 [0–0]	0.06	0 [0–150]	0.32
Colloids (ml)	1000 [563–1000]	500 [0–1500]	0.40	1000 [500–1625]	0.83
Crystalloids (ml)	3000 [2350–3875]	2725 [2075–3500]	0.80	3400 [2500–4125]	0.57
Norepinephrine (%)	50	75		90	
Hemoglobine (g/dl)	10.8 [9.2-12.6]	10.9 [9.9-12.3]	0.73	11.4 [9.3-12.4]	0.74
StO_2_ - baseline (%)	86 [83–90]	86 [81–90]	0.68	81 [78–84]	0.01
StO_2_ - average (%)	83 [77–87]	85 [78–91]	0.45	78 [74–82]	0.07
StO_2_ - minimum (%)	71 [59–79]	76 [70–82]	0.22	70 [57–72]	0.40
ScvO_2_ - baseline (%)	80 [70–85]	81 [75–84]	0.91	75 [74–83]	0.53
ScvO_2_ - average (%)	77 [70–81]	81 [77–83]	0.09	78 [72–83]	0.35
ScvO_2_ - minimum (%)	61 [55–68]	70 [59–72]	0.13	65 [57–72]	0.38

Dobu ^+^, subgroup of intervention subjects that did receive dobutamine; COPD, chronic obstructive pulmonary disease; CAD, coronary artery disease; CHF, chronic heart failure; IDDM, insulin dependent diabetes mellitus; TIA, transient ischemic attack; AAA, abdominal aortic aneurysm; RBC, red blood cell; StO_2_, tissue oxygenation; ScvO_2_, central venous oxygen saturation; baseline: start surgery; *Control versus Intervention; ^§^ Control versus Dobu^+^.

### Tissue oxygenation

Baseline StO_2_ was 85 [82–90]% in all patients. In the control group (n = 20) the median StO_2_ value was 83 [77–87]%. In eleven of these cases StO_2_ values showed a decline below the threshold of 80%. Median StO_2_ values were < 80% in seven patients in the control group.

**Figure 3:**
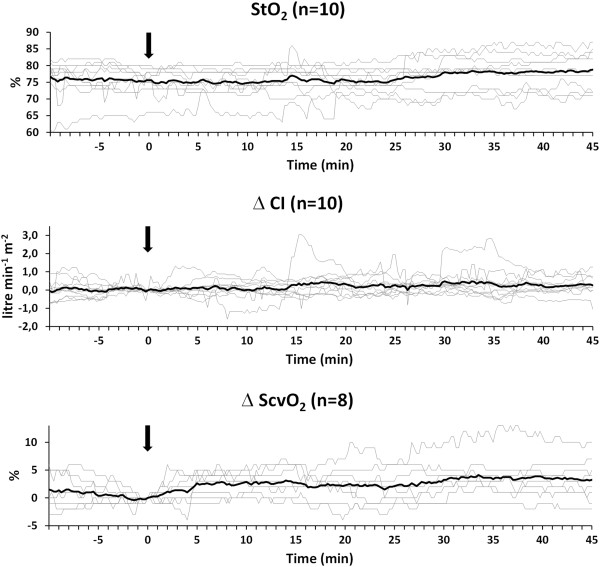
**Evolution of the individual patient values (thin lines) and the average values (thick line) of the tissue oxygenation (StO**_**2**_ **) and the relative change of both cardiac index (CI), delta CI, and central venous oxygen saturation (ScvO**_**2**_ **), delta ScvO**_**2**_ **(data of two patients missing/inadequate due to technical problems).** All graphs are synchronized at the moment of the first dobutamine administration (Time = 0; arrow). Values are shown from 10 minutes before dobutamine administration until 45 minutes thereafter.

No difference was found between the median intraoperative StO_2_ values of the control group and intervention group (83 [77–86.5]% vs. 82.5 [78–90]%; p = 0.83). In half of the patients randomized for the intervention group StO_2_ values showed a drop below the predefined threshold of 80%. Based on the study protocol dobutamine was administrered in those ten cases (Dobu^+^ subgroup; Table 1). Maximum median dobutamine dose was 4.9 [4.3-7.0] μg kg^-1^ min^-1^. The predefined maximum dose of 12.5 μg kg^-1^ min^-1^ was never reached. In one case dobutamine dosage was limited (10 μg kg^-1^ min^-1^) since elevation in heart rate was considered too high (>100 beats per minute) by the attending anesthesist. Figure 3 shows the graphs of the continuously measured StO_2_ values in the Dobu^+^ subgroup. Dobutamine induced a 6 [-3 to 10]% increase in StO_2_, a 0.3 [0.0-0.6] litre min^-1^ m^-2^ increase in CI, and a 4 [0 to 7]% increase in ScvO_2_ (Figure 3).

In the control group 9 patients (3/9 aorta surgery) received red blood cell (RBC) transfusions (range 300–3400 ml). Three patients from the intervention group (3/3 aorta surgery) received RBC transfusions (range 600–1800 ml). Two patients in the Dobu^+^ subgroup received 600 ml RBC transfusions. There was no significant difference between groups in either the amount of RBC transfused (p = 0.06 and p = 0.32, respectively), in number of patients who received RBC transfusions (p = 0.08 and p = 0.25, respectively) or in post transfusion hemoglobine values (p = 0.73 and p = 0.74, respectively); Table 1.

### Outcome data

Neither the total amount of complications nor the distribution of complications showed significant differences between groups (Table 2). Eleven patients in the intervention group had no complication, versus 7 in the control group (p = 0.20). Also, the median length of stay in various wards and hospital stay were equally distributed. One patient randomized to the intervention group died within 28 days postoperatively due to bowel ischemia noted during relaparotomy. One relaparotomy in the control group revealed leakage after pancreatic surgery.

**Table 2:** **Postoperative outcome**

	Control (n = 20)	Intervention (n = 20)	P-value*	Dobu ^+^ (n = 10)	P-value ^§^
Number of complications (n)	20	11	0.23	7	0.49
Patients without complications (n)	7	11	0.34	4	1.00
Complications			0.35		0.56
Pneumonia	4	5		3	
Respiratory failure	1				
Cardiac failure		1		1	
Supraventricular arrhythmia	3	1			
Relaparotomy	1	1		1	
Ileus	3	1		1	
Urinary tract infection	2	1		1	
Renal failure	1				
TIA	1				
Delirium	4				
Hematoma	1				
Death		1			
SOFA score	5 [2-12]	6 [5-11]	0.73	5 [5-9]	0.78
TISS score	25 [21–33]	26 [22–30]	0.95	28 [26–48]	0.96
LOS ICU (hours)	22 [21–45] (n = 15)	22 [17–38] (n = 15)	0.53	22 [17–43] (n = 8)	0.50
LOS PACU (hours)	18 [17-22] (n = 5)	18 [13-22] (n = 5)	0.75	17 [17,18] (n = 2)	0.42
LOS FFD (hours)	18 [8–37]	17 [6-23]	0.39	18 [13–36]	0.70
LOS Hosp (days)	12 [9-17]	13 [7-17]	0.96	15 [8-25]	0.72

Dobu^+^, subgroup of intervention subjects that did receive dobutamine; SOFA, sequential organ failure assessment; TIA, transient ischemic attack; LOS, length of stay; ICU, intensive care unit; PACU, post anesthesia care unit; FFD, fit for discharge; Hosp, hospital; *Control versus Intervention; ^§^ Control versus Dobu^+^.

Based on the average number of postoperative complications between the control and intervention group, a sample size was calculated revealing at least 118 patients (59 per group) will be needed to gain significant results in a follow-up study, with a power of 0.80.

### Feasibility

The overall protocol adherence was 96% in the control group and 92% in the intervention group (Figure 2). Protocol adherence was determined by assessing the proportion of patients that had to be excluded due to protocol violation and deviation and consequently assessing the number of cases where the protocol was successfully followed. The total exclusion amounted to 20% (17% from the control group and 23% from the intervention group) but exclusion due to protocol deviation or violation was only 6%.

## Discussion

During (high-risk) surgery or during treatment of critically ill patients, the ultimate goal is to maintain or to restore sufficient StO_2_ since it is a prerequisite of aerobic metabolism. In recent years the clinical use of near infrared spectroscopy (NIRS) to detect tissue oxygenation has increased, especially in the monitoring of critically ill patients and during cardiac surgery [8–10]. Data on the intraoperative use of StO_2_ in non-cardiac surgery however are scarce. Of note, the study designs differ substantially, which hampers proper comparison of the results. A randomised controlled trial excecuted in a critical care unit showed that postoperative application of stroke volume guided therapy and low-dose inotropic therapy (dopexamine) was associated with improved tissue oxygenation [11]. However, due to study design and insufficient power the investigators were unable to demonstrate an intervention-related reduction in complications rates. Other recent observations revealed that minimum StO_2_ values in the perioperative setting are inversely associated with outcome [4]. These findings suggest that perioperative optimisation of StO_2_ values may result in better outcome. The present results cannot fully confirm the abovementioned studies. Despite the category of patients randomised here (all ASA III, age > 65 yr) who are at elevated risk for lower StO_2_ values [12] and perioperative morbidity [13] a significant reduction in the number of complications in the intervention group could not be demonstrated in this pilot study. The relatively small number of patients is probably the main reason for this. Another important factor is the fact that only half of the patients randomized for the intervention group actually required dobutamine. Also, in only 40% of the patients who received dobutamine the predefined StO_2_ threshold of 80% was reached permanently. One could argue if dobutamine administration was the most favorable intervention. The increase in myocardial contractility (β_1_ adrenoceptors) and peripheral vasodilation (β_2_ adrenoceptors) generated a modest increase in StO_2_, CI and ScvO_2_.

Our observations raise another relevant issue, namely the adequacy of the predefined StO_2_ threshold of 80% used in this study. StO_2_ values below 80% are considered inadequate [10, 14] and together with recommendations of the manufacturer a threshold of 80% seemed reasonable. However, in patients scheduled for non-cardiac surgery, the time averaged StO_2_ values of approximately 85% have been described [4, 12]. These StO_2_ values are comparable to values seen in healthy volunteers breathing roomair [14, 15]. Hence, the StO_2_ threshold used in the present study may have been insufficient and the use of a higher StO_2_ threshold may have led to fewer complications in the intervention group. Abovementioned studies did not describe an optimal StO_2_ target and therefore another StO_2_ threshold than 80%, i.e. 85%, may be considered for future studies. Of note, flipside to that coin may be increased use of fluids and vasoactive medication.

We observed a trend of an increased number of RBC transfusion and of more patients in whom RBC transfusion were conducted in the control group compared to the intervention group. Although RBC transfusions were not controlled by study protocol other than a transfusion threshold of Hb = 5.0 mmol/L we doubt if the transfusions conducted were of influence on our results. Surely, the effect of RBC transfusions on tissue oxygenation by increasing oxygen delivery is yet unclear [16, 17]. RBC transfusions elevate the hemoglobin content in the microcirculation but not necessarily the microcirculatory perfusion in cardiac surgery patients [18]. However, considerable interindividual variability exists and the effects of RBC transfusion on StO_2_ values seem unpredictable [19, 20].

A yet unraveled issue with application of NIRS is the choice of measurement site [21]. For the measurement of peripheral tissue oxygenation multiple measurement sites are available. However, only few sites including the thenar eminence are established [10]. The thenar eminence has a relatively thin fat layer with only minor inter-individual variation. Also, the thenar eminence participates in systemic edema formation to a lesser extent than other skin regions [22] Abovementioned arguments together with the practical perspective from an anesthesists viewpoint explain our choice for the thenar eminence as measurement site. Nevertheless, in general we suspect that StO_2_ measurements mostly convey information on microcirculatory integrity. It is possible that only after a persisting and sufficiently large change in systemic oxygen balance is reflected in thenar StO_2_. The main research question is whether the goal-directed StO_2_ based treatment is correct and well dosed. Based on the present study we are unable to answer that question.

The development of a feasible protocol to determine any significant differences between the study groups remains important. For a study as such where small differences between study groups are observed, it is essential to accurately collect data. The study protocol should be clearly outlined for investigators, as to avoid protocol deviations. We believe that the present study fulfills these criteria. Data were collected continuously, the attending anesthesists were informed comprehensibly and the study protocol was present in the operating room at all times. Studies have shown that protocol adherence becomes one of the main barriers when implementing changes in a clinical setting, with regards to goal directed therapy (GDT) [23–25]. Reasons for low adherence to GDT include time consumption of conducting the protocol, the tendency of monitoring to be invasive and personal disbelief that such a protocol will result in better outcome [25]. Protocol adherence in this study was 94% which is considered acceptable (≥75%) [2]. This can be attributed to the convenient and non-invasive nature and easy interpretation of the obtained data provided by the NIRS equipment [10].

This study has several limitations. First, the observations of the present randomized controlled pilot study are limited to the intraoperative period only. One could argue that the direct postoperative hours are as interesting in efforts to improve outcome after high-risk surgery. Indeed, clinically important fluctuations in the balance between DO_2_ and VO_2_ do also occur in the first postoperative hours [26, 27] and corresponding hemodynamic interventions seem appropriate. On top of that, postoperative hemodynamic optimisation improves tissue oxygenation [11] and we believe that goal-directed protocols ideally should also cover the early postoperative period. However, because of technical challenges in reliably transporting all the equipment, including the data logging devices, along with the patient to the ICU or PACU, measurements were restricted to the intraoperative period. While our results tend to indicate an advantage of StO_2_ based goal-directed therapy, the limited power of this pilot restricts any clear recommendations before further larger studies are conducted.

## Conclusions

In conclusion, this is the first randomized controlled study that used StO_2_ values as a target for hemodynamic optimization in high-risk abdominal surgery patients. Our pilot results are inconclusive on the perioperative optimisation of StO_2_ values in relation to outcome after high-risk abdominal surgery. Nevertheless, the perioperative strategy described deserves emulation and further research is obligatory to define both the optimal StO_2_ threshold and intervention to treat or prevent tissue hypoperfusion.

## Abbreviations

BIS: Bispectral monitoring
C_e_t: Effect site concentration
CI: Cardiac index
CO: Cardiac output
CVP: Central venous pressure
DO_2_: Oxygen delivery
FFD: Fit for discharge
ICU: Intensive care unit
NIRS: Near infrared spectroscopy
PACU: Post-anesthesia care unit
RBC: Red blood cell
rSO_2_: Regional cerebral oxygenation
ScvO_2_: Central venous oxygen saturation
SpO_2_: Oxygen saturation
StO_2_: Tissue oxygenation
TCI: Target-controlled infusion.
